# Microbial Diversity in the Rhizosphere Soils of Three Different Populations of *Paphiopedilum helenae*, a Critically Endangered Wild Orchid

**DOI:** 10.3390/microorganisms13102282

**Published:** 2025-09-30

**Authors:** Kanghua Xian, Jinhan Sang, Jiang Su, Ningzhen Huang, Wenlong Wu, Jinxiang He, Baojun Liu, Chuanming Fu

**Affiliations:** 1 Guangxi Key Laboratory of Plant Conservation and Restoration Ecology in Karst Terrain, Guangxi Institute of Botany, Guangxi Zhuang Autonomous Region and Chinese Academy of Sciences, Guilin 541006, China; 2 School of Life and Environmental Sciences, Guilin University of Electronic Technology, Guilin 541004, China

**Keywords:** *Paphiopedilum helenae*, rhizosphere microorganism, geographic differentiation, endangered wild plants

## Abstract

In the Red List of Threatened Species, released by International Union for Conservation of Nature (IUCN), *Paphiopedilum helenae* has been classified as an endangered species. It exhibits exceptional decorative value and germplasm resource potential. To elucidate the ecological adaptation of this species and the characteristics of its rhizosphere microbiome, bacterial 16S rRNA and fungal ITS sequences of three wild populations of *P. helenae* were investigated using Illumina high-throughput sequencing technology and the microbial community structures and diversities were systematically compared. These three populations were spanned across distinct geographical locations in Longzhou County, Guangxi. The results showed that the bacterial community in the rhizosphere soil of *P. helenae* comprised 31 phyla, primarily including *Actinobacteriota*, Proteobacteria, *Chloroflexi* and *Acidobacteriota*. On the other hand, the fungal community consisted of 10 phyla, dominated by *Ascomycota* and *Basidiomycota*. There were significant differences in the diversity of rhizosphere microbes across different populations of *P. helenae*. The LG population had the highest bacterial richness (Chao index: 2912.71 ± 131.73; *p* < 0.05) and diversity (Shannon index: 6.40 ± 0.06; *p* < 0.01), while the MQ population had the lowest diversity (Shannon index: 3.47 ± 0.24; *p* < 0.01) of fungi. The degree of variation in fungal β-diversity was significantly higher than that of bacteria. Soil organic matter (SOM) and available nitrogen (AN) contents were the core factors shaping the microbial communities in the rhizosphere soil of *P. helenae*, which jointly explained 49.87% and 16.39% of variations in the bacterial and fungal communities. Furthermore, population-specific enrichment of functionally significant microorganisms was evident. Population MQ was enriched with plant growth-promoting and stress-resistant fungi, such as *Geminibasidium*, *Trichoderma*, etc. Population LG was enriched with oligotrophic bacteria (e.g., *Patescibacteria*), while population SL exhibited an overwhelming dominance of *Ascomycota* (93.25%) and enrichment of pathogenic fungal genus Nigrospora. This research revealed the variations in the functional adaptation strategy of *P. helenae* and the microbial communities in the rhizosphere soils across different geographical locations. This suggests that microbial community imbalance in rhizosphere soil may be one of the factors leading to the endangerment of this plant species. The study proposed a differentiated protection strategy for endangered plant species based on microbial resources. The results provide a theoretical basis for development of a “microorganism-assisted protection” strategy for ecological restoration and sustainable utilization of endangered orchid plants.

## 1. Introduction

*Paphiopedilum helenae* Aver. is a perennial orchid species, which was first discovered in Cao Bang Province of Vietnam, and its type specimen was formally described in 1996 [1]. In China, its presence in western Guangxi was confirmed by reports and official documents published in 2005 and 2007 [2]. This orchid species inhabits the impoverished karst limestone environments, primarily growing in narrow soil crevices on cliff faces near summits or as a lithophyte on rock walls. Severe habitat constraints, including soil infertility, karstic drought, as well as limited seed germination and shoot differentiation result in critical regeneration of restricted populations. As a result, this species has been classified as an endangered species that requires urgent conservation intervention. Despite its ecological significance, limited research has been conducted on its endangerment mechanisms and conservation biology due to extreme habitat specificity and scarce wild individual plants. Currently, *P. helenae* is classified as Endangered (EN) on the IUCN Red List. The species has been designated as a Grade I Nationally Protected Plant in China. Its conservation is prioritized in both the National Project for Rescuing Extremely Small Populations of Wild Plants and the Guangxi Implementation Plan for Rescuing Extremely Small Populations, as one of the most endangered species requiring urgent conservation intervention. Moreover, *P. helenae* is one of the most extraordinary ornamental orchids, renowned for its distinctive floral architecture, vibrant colors, and exceptionally prolonged blooming period, with an individual flower persisting for 1–2 months. Its wild germplasm is a valuable genetic resource for breeding novel ornamental hybrids [3]. Given the critically high extinction risk of *P. helenae*, implementing targeted research integrating rescue conservation and sustainable utilization strategies is imperative.

The rhizosphere microbiome plays a crucial role in plant growth, health and nutrient acquisition [4]. Studies have revealed that microbial communities in the rhizosphere harbor abundant stress-resistant and growth-promoting microbial resources, including fungi such as *Trichoderma*, *Aspergillus*, and *Penicillium* genera, as well as bacteria like *Pseudomonas* and *Bacillus*. These beneficial microorganisms enhance growth of host plants through multiple mechanisms, such as promoting nutrient mineralization, regulating immune responses, and strengthening stress tolerance [5], thereby promoting the development of plant and its adaptation to extreme environments. Concurrently, plant pathogenic microbes colonize the rhizosphere, thereby breaching the protective microbial barriers and overcoming plant’s innate defenses to cause disease [6]. Consequently, the rhizosphere is a complex ecosystem. Understanding its microbial community structure, functional dynamics, and regulatory mechanisms is essential for deciphering plant-microbe interactions and developing novel strategies to enhance the growth and environmental adaptability of plants. This knowledge is particularly significant for conservation initiatives targeting rare and endangered orchid species.

Current research across diverse plant systems has confirmed the pivotal role of rhizosphere microorganisms in soil nutrient cycling and plant health [7,8,9]. For conservation and utilization of *Paphiopedilum* orchids, investigations into rhizosphere microbiomes are gaining increasing attention. Tian et al. [10] utilized ITS high throughput sequencing to characterize the fungal communities in the rhizosphere soils of three critically endangered orchids, including *P. armeniacum*, *P. wenshanense*, and *P. emersonii*. The findings revealed species-specific dominant fungal taxa, which primarily included saprotrophs and ectomycorrhizal fungi. Notably, the well-coordinated symbiotic interactions among these fungi enhanced the adaptation of these orchids to challenging environmental conditions. In a complementary study, Tian et al. [11] examined the habitat ecology and fungal communities in the rhizosphere soils of *Subgenus Paphiopedilum* species. Significant differences in the fungal community structure were observed among different habitats, with *Basidiomycota* and *Ascomycota* being the dominant phyla. Functional analysis revealed symbiotic and saprotrophic fungi as primary functional guilds. LEfSe (Linear discriminant analysis effect size) analysis further demonstrated that the habitat preferences of *Subgenus Paphiopedilum* species were reflected in their distinct fungal community assemblages. Wu [12] investigated the rhizosphere soil of seven *Subgenus Paphiopedilum* species and reported *Ascomycota* and *Basidiomycota* as dominant fungi, while Actinobacteria and Proteobacteria were dominant in the bacterial communities. Soil physicochemical factors were identified as the key drivers of microbial community composition and diversity.

Although significant progress has been made in research on the rhizosphere microorganisms related to some *Paphiopedilum* species, studies focusing on the rare species like *Paphiopedilum helenae* remain limited. Existing literature has only documented a comparative study conducted by Zhang Zhenliang [13] on the rhizosphere microorganisms in four extremely small populations of *Paphiopedilum*, including *P. helenae.* There is no systematic research that specifically targeted different geographical populations of *P. helenae.* Currently, *P. helenae* is distributed across five sites in China. Among these, three distribution sites are in Longzhou County, Guangxi, hosting relatively larger wild populations of *P. helenae*. These populations exhibit a terrestrial growth pattern with occasional lithophytic growth, allowing the collection of rhizosphere soil. Both the habitat conditions and population structures in these sites are highly representative. On the other hand, populations in Jingxi City and Napo County are smaller and primarily lithophytic, with roots exposed on rock surfaces, making it difficult to collect the samples of rhizosphere soil. Given the harsh habitats and geographic isolation between *P. helenae* populations, this study primarily addresses the following questions: Do significant differences exist among distinct populations inhabiting a typical karst habitat (Longzhou County, Guangxi) in terms of composition, structure, and diversity of rhizosphere bacterial and fungal communities? Are these differences associated with specific microenvironmental factors? Do the rhizosphere microbial communities harbor unique functional groups or core members potentially aiding host adaptation to extreme stressors (e.g., drought, nutrient poverty)? To address these questions, three populations of *P. helenae* in Longzhou County were selected as the research subjects. Using Illumina high-throughput sequencing technology, the variable regions of bacterial 16S rRNA and fungal ITS genes in rhizosphere soils were sequenced to systematically analyze the structure and diversity patterns of rhizosphere bacterial and fungal communities. The findings of this study will shed light on the ecological adaptation mechanisms of this endangered species by providing microbial ecological insights. The study will offer theoretical support for developing scientific conservation strategies and sustainable utilization plans for *P. helenae*.

## 2. Materials and Methods

### 2.1. Soil Sample Collection and Processing

**Figure 1:**
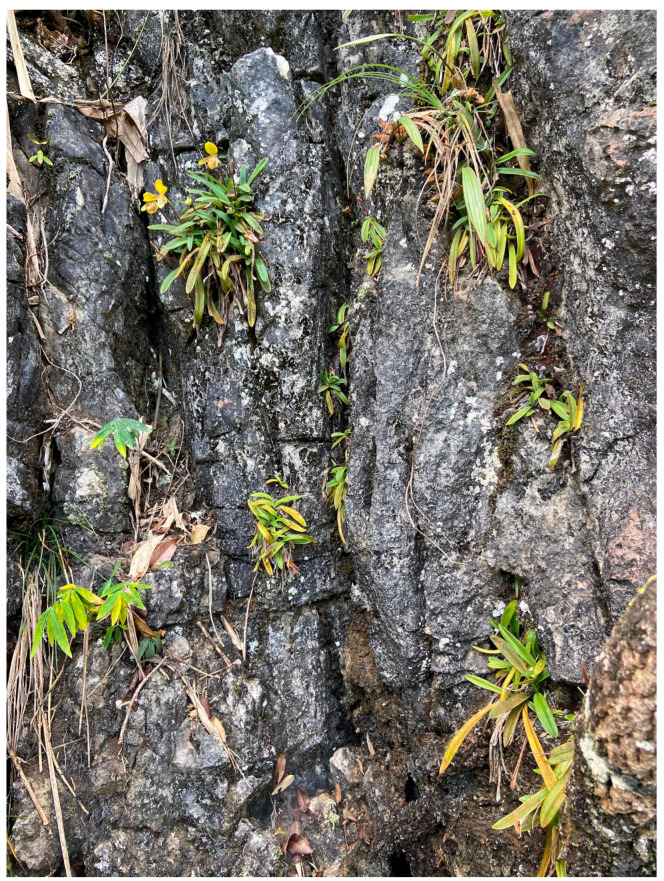
The growth situation of *Paphiopedilum helenae* in its natural habitat.

Three wild populations of *Paphiopedilum helenae* in Longzhou County, Guangxi, were designated as the MQ, LG, and SL populations (Table 1). Figure 1 showed the photos of *P. helenae* in its natural habitat. Following the principle of random multi-point mixing, five healthy and well-growing *P. helenae* individuals were selected from each population. To specifically target the root-microbe interface, we collected the tightly adhering soil fraction (0–0.5 cm) from the root surface, which represents the rhizoplane and immediate rhizosphere compartment, using a soft brush as described by Zheng et al. (2024) [14]. This method is standard for orchid studies to minimize bulk soil contamination and focus on the microbiome most influenced by root exudates. The rhizosphere soil samples collected from these five plants were then thoroughly mixed in equal amounts to form a representative composite sample. Three biological replicates were set up for each population (i.e., a total of 15 plants sampled per population). To avoid impacting plant growth, the number of roots sampled per plant did not exceed one-third of the total root system [15]. The composite soil from each population was placed into sterile zip-lock bags, labeled with sampling information, and immediately transported to the laboratory in a portable cooler. Collected soil samples were stored at −80 °C for subsequent DNA extraction and sequencing of bacterial 16S rRNA and fungal ITS regions. The field work and soil sampling procedures were conducted in compliance with local regulations. The procedures were approved under two permits: (i) No. Nongbanhan [2022] No. 1, issued by the Management Center of Guangxi Nonggang National Nature Reserve, Chongzuo City, and (ii) No. Guilin Shenzhunbao [2022] No. 4, issued by the Forestry Bureau of Guangxi Zhuang Autonomous Region. All effort were made to minimize disturbance to the natural habitat and the resident population of *P. helenae*.

### 2.2. Analytical Methods

Total genomic DNA of soil microorganisms was extracted using the soil genomic DNA rapid extraction kit (Sangon Biotech Co., Ltd., Shanghai, China). The extracted DNA samples were shipped on dry ice to Shanghai Majorbio Bio-Pharm Technology Co., Ltd. (Shanghai, China) for sequencing. Sequencing was performed on the Illumina MiSeq PE300 platform. The workflow included PCR amplification, pooling and purification of PCR products, and library construction.

For bacterial community analysis, the V3–V4 hypervariable regions of the 16S rRNA gene were amplified using primers 338F (5′-ACTCCTACGGGAGGCAGCAG-3′) and 806R (5′-GGACTACHVGGGTWTCTAAT-3′). The PCR conditions were as follows: initial denaturation at 95 °C for 3 min; followed by 28 cycles of 95 °C for 30 s, 53 °C for 30 s, and 72 °C for 45 s; with a final extension at 72 °C for 10 min. For fungal community analysis, the ITS1 region of the fungal rRNA gene was amplified using primers 338F (5′-ACTCCTACGGGAGGCAGCAG-3′) and 806R (5′-GGACTACHVGGGTWTCTAAT-3′). The PCR conditions were: initial denaturation at 95 °C for 3 min; followed by 37 cycles of 95 °C for 30 s, 53 °C for 30 s, and 72 °C for 45 s; with a final extension at 72 °C for 10 min.

### 2.3. Quality Control and Analysis of Microbiome Sequencing Data

Raw sequencing data underwent assembly and quality control to generate valid sequences. Then the optimized sequences were clustered into operational taxonomic units (OTUs) using UPARSE 7.1 with 97% sequence similarity level, with data rarefied to the minimum sequence depth. The OTU clustering workflow included: (1) extracted unique sequences from optimized reads to reduce computational redundancy; (2) filtered out singleton sequences; (3) clustered non-singleton sequences into OTUs at 97% similarity while removing chimeras to obtain representative sequences; (4) mapped all optimized sequences to OTU representatives and selected sequences with ≥97% similarity to construct the OTU table. Taxonomic analysis of OTU representatives (97% similarity) was performed using the RDP classifier Bayesian algorithm, with community composition statistically characterized across eight taxonomic levels: Domain, Kingdom, Phylum, Class, Order, Family, Genus, Species.

### 2.4. Analysis of Rhizosphere Soil Microbial Composition

Based on sequencing data, microbial community bar plots were generated using R language tools, while Alpha diversity indices (Ace, Chao, Simpson, and Shannon) were calculated via Mothur (v.1.30.2) software (https://www.mothur.org/wiki/Calculators, accessed on 14 May 2025) under randomized subsampling to assess community richness and diversity. Specifically, the Ace and Chao indices reflect community richness, whereas Simpson and Shannon indices characterize diversity, with higher Ace, Chao, Shannon values and lower Simpson values indicating greater species diversity. Venn diagrams quantifying shared and unique OTUs across samples were produced using R (v3.3.1). Formulas followed [16]:

(1) $$D_{\mathrm{sim}pson}=\frac{\sum_{i=1}^{S_{obs}}n_{i}(n_{i}-1)}{N(N-1)}$$

(2) $$H_{\mathrm{shan}non}=-\sum_{i=1}^{S_{obs}}\frac{n_{i}}{N}\ln\frac{n_{i}}{N}$$

Sobs denotes the observed OTU count; *n_i_* denotes the number of sequences in the *i*-th OTU; *N* denotes the total sequence count.

### 2.5. Analysis of Rhizosphere Soil Microbial Community Structure Differences

Microbial community composition was analyzed using Qiime software (http://qiime.org/install/index.html, accessed on 13 May 2025), where Principal Coordinates Analysis (PCoA) was employed to visualize similarities and differences in community structures between samples. Linear Discriminant Analysis (LDA) was performed using the LEfSe software (https://huttenhower.sph.harvard.edu/lefse/, accessed on 14 May 2025) based on taxonomic composition to identify communities that contributed significantly to sample differentiation, referred to as LDA Effect Size analysis for intergroup community differences. At the OTU level, distance-based redundancy analysis (db-RDA) was conducted with R (version 3.3.1) to examine the relationship between environmental factors and the distribution of microbial species. Variance partitioning analysis (VPA) was also performed using R (version 2.4.3) at the OTU level to assess the contribution of environmental factors to the variation in soil microbial community distribution. Additionally, a Spearman correlation heatmap analysis was carried out with R (version 3.3.1) to investigate the associations between environmental factors and the dominant phyla of bacteria and fungi.

### 2.6. Data Processing

Statistical significance was tested using one-way ANOVA with a significance level of 0.05, followed by multiple comparisons via the Least Significant Difference (LSD) method; all analyses were performed using IBM SPSS Statistics 21.0, while data organization and statistical computations were conducted in Microsoft Excel 2010.

## 3. Results

### 3.1. Physicochemical Properties of Paphiopedilum helenae Rhizosphere Soil

The physicochemical parameters of rhizosphere soil samples from distinct *P. helenae* populations are presented in Table 2. The soil pH ranged from slightly acidic to slightly alkaline (5.97–8.05), with a mean value of 7.33. The LG population exhibited the highest mean levels of nitrogen (N), phosohorus (P), available phosphorus (AP) and available potassium (AK). In contrast, the SL population showed the highest mean soil organic matter (SOM) and ammonium nitrogen (AN) content, while the MQ population contained the highest potassium (K) concentration.

### 3.2. Rhizosphere Soil Microbial Community Composition of Paphiopedilum helenae

#### 3.2.1. Bacterial Community Composition Analysis

**Figure 2:**
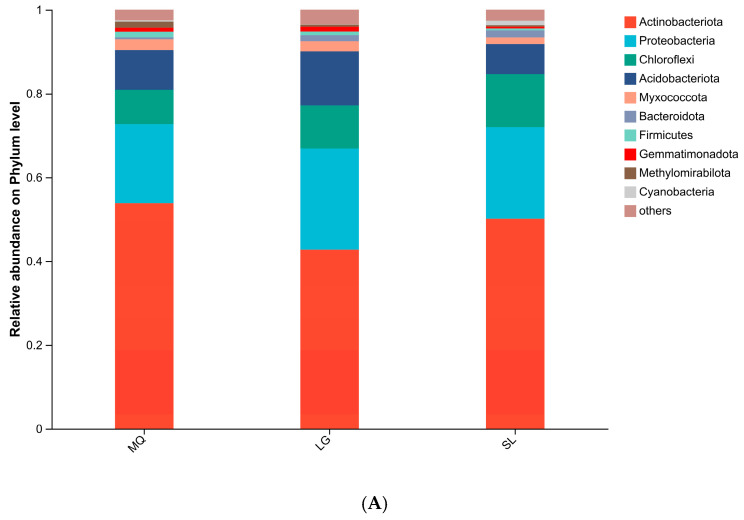

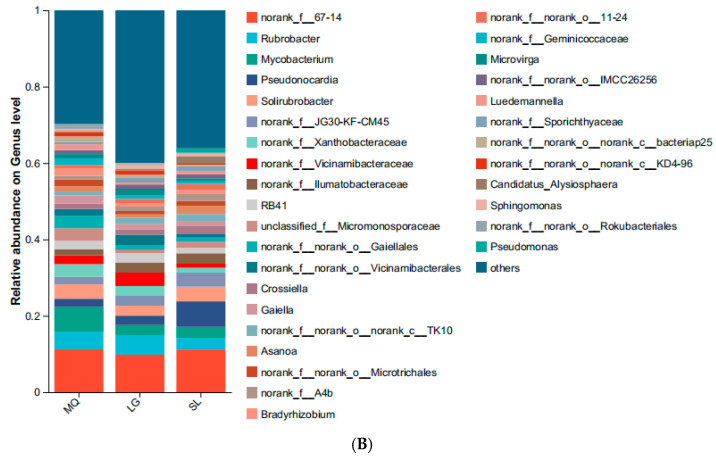
Relative abundance of soil bacteria at phylum (**A**) and genus (**B**) levels.

All samples yielded 472,169 valid sequences with lengths concentrated at 401–440 bp. Following rarefaction to the minimum sequence depth, bioinformatic analysis identified 4243 OTUs at 97% similarity, taxonomically assigned to 31 phyla, 90 classes, 232 orders, 350 families, 644 genera, and 1268 species. At the phylum level (Figure 2A), dominant bacterial phyla included *Actinobacteriota*, Proteobacteria, *Chloroflexi*, and *Acidobacteriota*, with *Actinobacteriota* and Proteobacteria collectively constituting >60% of total bacterial abundance. *Actinobacteriota* abundance peaked in the MQ population (53.82%) and was lowest in LG (42.77%), while Proteobacteria exhibited the opposite trend. At the genus level (Figure 2B), the major genera included norank-f-67-14 and Rubrobacter, where norank-f-67-14 reached its highest abundance in MQ (11.24%) and Rubrobacter reached its highest abundance in LG (5.17%).

#### 3.2.2. Fungal Community Composition Analysis

**Figure 3:**
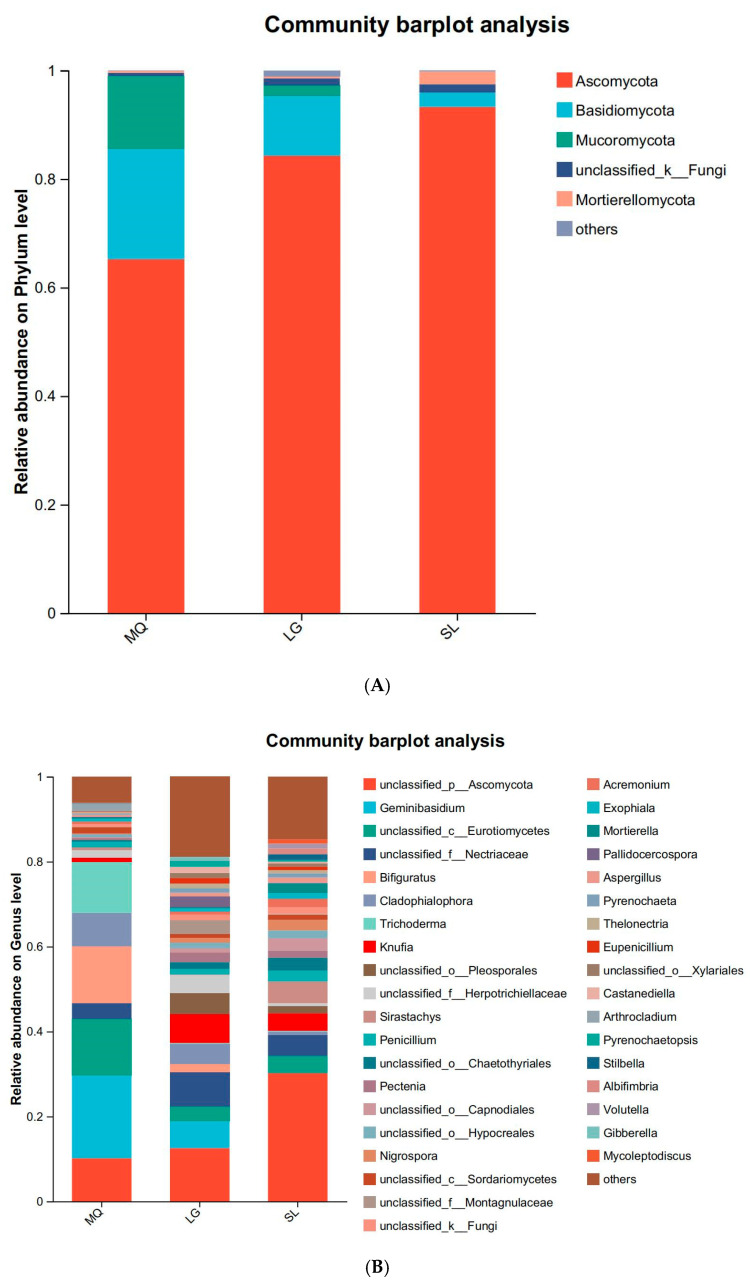
Relative abundances of soil fungi at phylum (**A**) and genus (**B**) levels.

All soil samples yielded 488,911 valid sequences with lengths concentrated at 200–300 bp. Following rarefaction to the minimum sequence depth, bioinformatic analysis identified 2496 OTUs at 97% similarity, taxonomically assigned to 10 phyla, 35 classes, 102 orders, 245 families, 498 genera, and 767 species. Dominant phyla (Figure 3A) included *Ascomycota* (absolute dominant; mean abundance: 80.93%), *Basidiomycota*, and *Mucoromycota*, with *Ascomycota* peaking in SL (93.25%). The MQ population exhibited significant enrichment of *Basidiomycota* (20.35%) and *Mucoromycota* (13.37%), while *Mucoromycota* was nearly undetected in SL. Fungal composition varied markedly among populations: *Geminibasidium* reached an abundance of 19.51% in MQ, significantly higher than in other samples (LG: 6.39%; SL: undetected), and was nearly absent in SL rhizosphere soil (Figure 3B). Additionally, *Bifiguratus* and *Trichoderma* abundances were markedly higher in MQ than other populations.

### 3.3. α-Diversity of Rhizosphere Soil Microorganisms in Paphiopedilum helenae

The abundance indices (Ace, Chao) and diversity indices (Shannon, Simpson) of bacterial and fungal communities in the rhizosphere soil of *Paphiopedilum helenae* are shown in Table 3. As seen in the table, the bacterial Ace and Chao indices in the rhizosphere soil followed the order LG > MQ > SL, indicating that the bacterial richness was highest in the LG population rhizosphere soil. Significance analysis revealed that the bacterial richness in the LG rhizosphere soil was significantly higher than in the other samples, while there was no significant difference between the MQ and SL populations. The Shannon and Simpson indices showed that bacterial diversity in the LG population was significantly higher than in the MQ and SL populations, with no significant difference between the MQ and SL populations.

The fungal Ace and Chao indices followed the order LG > SL > MQ, indicating that fungal richness was highest in the LG population rhizosphere soil. Fungal richness showed no significant difference between the LG and SL populations, while it was significantly lower in the MQ population compared to the LG and SL populations. The Shannon and Simpson index results showed that fungal diversity in the MQ population rhizosphere soil was significantly the lowest, with no significant difference between the LG and SL populations.

### 3.4. Correlation Analysis of Rhizosphere Soil Microbial Communities in Paphiopedilum helenae

**Figure 4:**
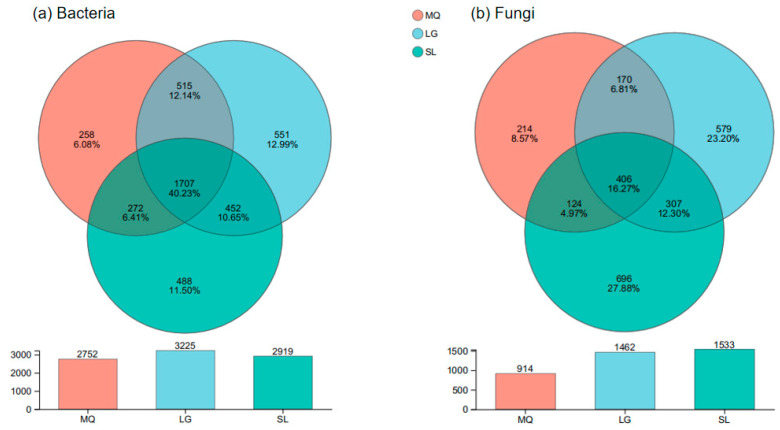
Venn diagram of bacteria and fungi in rhizosphere soil of different populations of *Paphiopedilum helenae*.

Venn diagrams depicting OTUs are shown in Figure 4. From Figure 4a, it can be observed that the LG population had the highest number of bacterial OTUs (3225), while the MQ population had the fewest (2752). The three populations shared 1707 bacterial OTUs, accounting for 40.23% of the total bacterial OTUs. The LG population had the highest number of unique bacterial OTUs (551, 12.99% of total OTUs), while the MQ population had the fewest unique bacterial OTUs (258, 6.08% of total OTUs). Figure 4b shows that the SL population had the highest number of fungal OTUs (1533), while the MQ population had the fewest (914). The three populations shared 406 fungal OTUs, accounting for 16.27% of the total fungal OTUs. The SL population had the highest number of unique fungal OTUs (696, 27.88% of total OTUs), while the MQ population had the fewest unique fungal OTUs (214, 8.57% of total OTUs). Combined analysis of the bacterial and fungal OTU Venn diagrams revealed that the degree of β-diversity variation in the fungal communities of the *P. helenae* rhizosphere was significantly greater than that in the bacterial communities, indicating that the variation in fungal communities across different populations was greater than that in bacterial communities.

**Figure 5:**
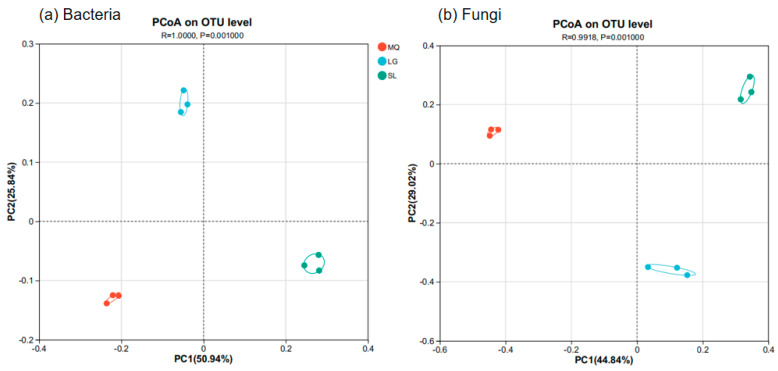
Principal co-ordinates analysis based on OUT ((**a**), Bacteria; (**b**), Fungi).

Principal Coordinates Analysis (PCoA) was used to explore differences in soil microbial community composition among the *P. helenae* populations, with results shown in Figure 5. PCoA analysis of both bacteria and fungi, coupled with ANOSIM (Analysis of Similarities, *p* = 0.001), showed significant differences in the composition of both bacterial and fungal communities among the populations. Furthermore, the differences between groups were significantly greater than the differences within groups.

### 3.5. Analysis of Microbial Species Differences in Paphiopedilum helenae Rhizosphere Soil

**Figure 6:**
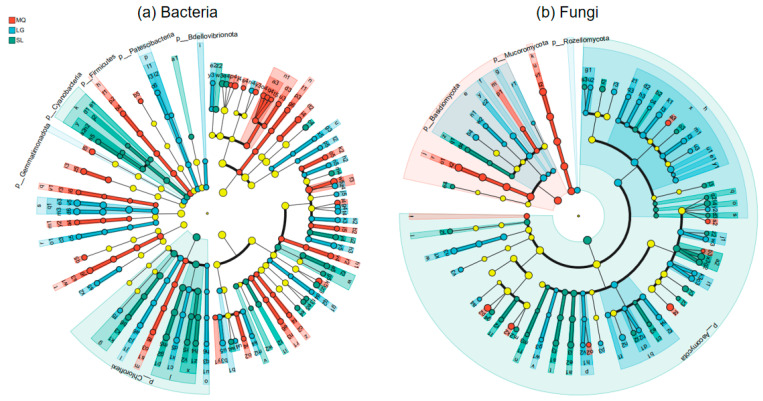
LDA effect size of differentially abundant groups.

Microorganisms identified based on LDA Effect Size (LEfSe) inter-group difference analysis, which were significantly enriched in the rhizosphere soil of *Paphiopedilum helenae* and significantly influenced inter-group differences, are shown in Figure 6. From the figure, it can be observed that at the bacterial phylum level, microbial taxa significantly contributing to inter-group differences included Firmicutes, *Gemmatimonadota*, *Patescibacteria*, *Bdellovibrionota*, *Chloroflexi*, and Cyanobacteria. Among these, Firmicutes was significantly enriched in the MQ population, *Gemmatimonadota*, *Patescibacteria*, and *Bdellovibrionota* were significantly enriched in the LG population. *Chloroflexi* and Cyanobacteria were significantly enriched in the SL population. At the bacterial genus level, significantly enriched species in the MQ population that contributed notably to inter-group differences included taxa like norank-f-*Xanthobacteraceae*. The LG population showed significant enrichment of bacteria such as norank-f-*Xanthobacteraceae*. The SL population exhibited significant enrichment of bacteria including uncultured-bacterium-g-*Pseudonocardia*. At the fungal phylum level, microbial taxa significantly influencing inter-group differences included *Ascomycota*, *Basidiomycota*, *Mucoromycota*, and *Rozellomycota*. Among these, *Basidiomycota* and *Mucoromycota* were significantly enriched in the MQ population. *Rozellomycota* was significantly enriched in the LG population, and *Ascomycota* was significantly enriched in the SL population. At the genus level, significantly enriched species in the MQ population that contributed notably to inter-group differences included *Geminibasidium*, *Geminibasidium* sp., *Bifiguratus*, *Bifiguratus* sp., *Trichoderma*, *Cladophialophora* sp., and *Cladophialophora*. The LG population showed significant enrichment of species such as *Montagnulaceae* sp. and *Pectenia*. The SL population exhibitedsignificant enrichment of species including *Sirastachys*, Nigrospora, and *Penicillium*.

### 3.6. Relationship Between Environmental Factors and Microbial Community Composition

**Figure 7:**
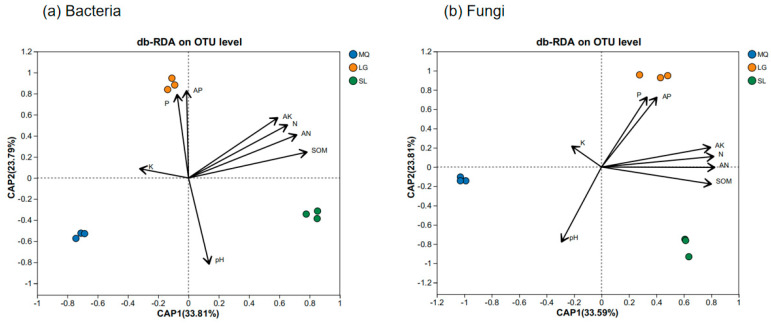
db-RDA of microbial community and environmental factors in rhizosphere soil of *Paphiopedilum helenae*.

**Figure 8:**
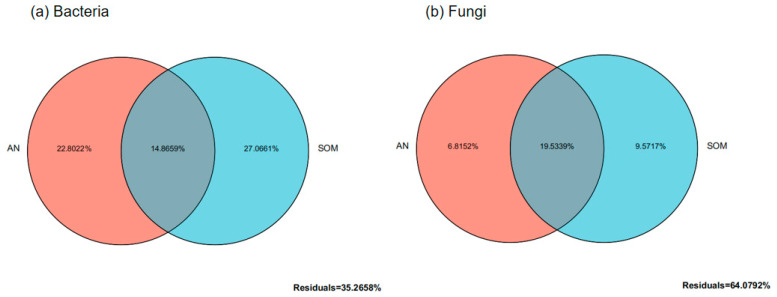
The contribution of environmental factors to the distribution of soil microbial communities.

Distance-based redundancy analysis (db-RDA) of environmental factors and microbial species distribution in *Paphiopedilum helenae* rhizosphere soil (Figure 7) revealed that for bacterial communities, CAP1 and CAP2 explained 33.81% and 23.79% of total variance respectively, with soil organic matter (SOM), available nitrogen (AN), total nitrogen (N), and available potassium (AK) exerting substantial influence. The db-RDA (distance-based redundancy analysis) results for the fungal community and environmental factors revealed that CAP1 and CAP2 explained 33.59% and 23.81% of the total variance respectively, with AN, SOM, N, and AK being key determinants. Furthermore, variance partitioning analysis (Figure 8) demonstrated that SOM and AN explained 27.07% and 22.80% of bacterial community variation respectively, establishing them as dominant drivers of bacterial dynamics, while for fungal communities, SOM and AN accounted for 9.57% and 6.82% of variation respectively, confirming their significant role in shaping fungal community structure.

### 3.7. Correlations Between Dominant Microorganisms and Ecological Factors

**Figure 9:**
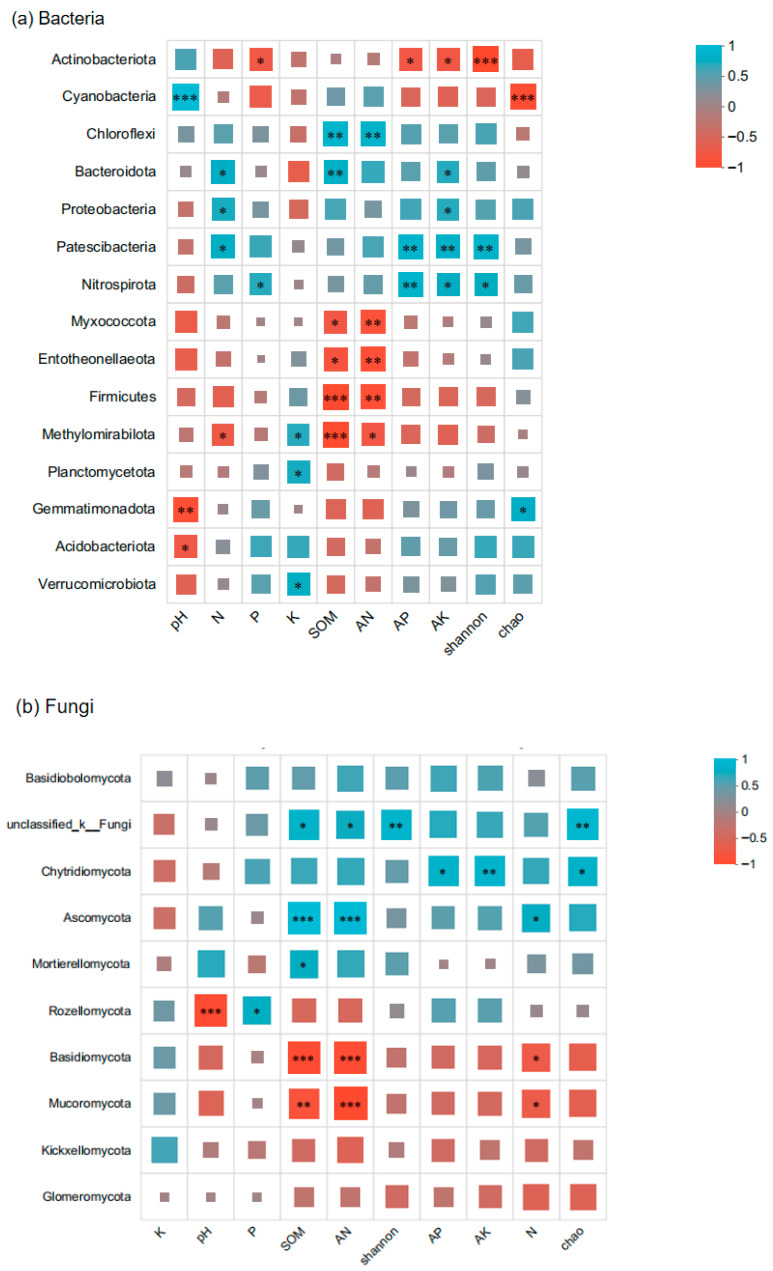
Spearman correlation between rhizosphere microbial community and ecological factors of *Paphiopedilum helenae.* The X-axis and Y-axis represent the environmental factors and species, respectively. R and *p* values were obtained through calculation. The R values are displayed with different colors in the graph, with a color gradient legend for different R values shown on the right. Statistical significance is indicated by asterisks: * for 0.01 < *p* ≤ 0.05, ** for 0.001 < *p* ≤ 0.01, and *** for *p* ≤ 0.001.

Soil factors demonstrated significant associations with *Paphiopedilum helenae* rhizosphere microbial communities, with differential influences observed across ecological parameters. The Spearman correlations between rhizosphere microbial communities of *Paphiopedilum helenae* and ecological factors are shown in Figure 9, SOM and AN exhibited positive mutual correlation, jointly enhancing *Chloroflexi* abundance while significantly (*p* < 0.05) or highly significantly (*p* < 0.001) reducing abundances of *Myxococcota*, Entotheonellaeota, Firmicutes, and *Methylomirabilota*; Soil pH showed significant or highly significant negative correlations with *Acidobacteriota* and *Gemmatimonadota*, respectively, while demonstrating a highly significant positive correlation with Cyanobacteria; N significantly increased abundances of *Bacteroidota*, Proteobacteria, and *Patescibacteria* while reducing *Methylomirabilota*; P significantly decreased *Actinobacteriota* but increased *Nitrospirota*; Kelevated abundances of *Methylomirabilota*, Planctomycetota, and Verrucomicrobiota; AP and AK showed positive synergy, jointly reducing *Actinobacteriota* while enhancing *Patescibacteria* and *Nitrospirota* with significant to highly significant effects. Additionally, the bacterial Shannon index correlated highly significantly negatively with *Actinobacteriota* but positively with *Nitrospirota* and *Patescibacteria*. The Chao index exhibited highly significant negative correlation with Cyanobacteria but significant positive correlation with *Gemmatimonadota*. SOM and AN also exhibited positive synergistic effects on fungal communities, jointly and significantly increasing the abundance of unclassified-k-Fungi while highly significantly (*p* < 0.01) enhancing *Ascomycota* abundance, but exerting highly significant negative impacts on *Basidiomycota* and *Mucoromycota*. Furthermore, pH exerted a highly significant inhibitory effect on the growth of *Rozellomycota*, whereas phosphorus (P) significantly increased *Rozellomycota* abundance. Positive correlation between AP and AK significantly to highly significantly elevated Chytridiomycota abundance. Nitrogen (N) significantly promoted *Ascomycota* while inhibiting *Basidiomycota* and *Mucoromycota*. Furthermore, the fungal Chao index showed significant to highly significant positive correlations with unclassified-k-Fungi and Chytridiomycota, and the Shannon index exhibited highly significant positive correlation with unclassified-k-Fungi, collectively indicating that SOM, AN, AP and AK significantly influence both fungal richness and diversity.

## 4. Discussion

### 4.1. Microbial Community Composition in the Rhizosphere Soil of P. helenae

Plants actively shape the microbiomes in their rhizosphere [17], and this study provides the first systematic revelation of rhizosphere microbial community structures and their influencing factors across geographically distinct populations of the critically endangered *P. helenae*. The rhizobacterial communities were predominantly composed of *Actinobacteriota*, Proteobacteria, *Chloroflexi*, and *Acidobacteriota*, while fungal co consistent with the findings reported for the rhizosphere soils of other *Paphiopedilum* species [11,12,13]. Orchid rhizospheres typically harbor bacterial phyla, such as Proteobacteria, *Acidobacteriota*, *Actinobacteriota* [18,19], etc., and fungal phyla like *Basidiomycota* and *Ascomycota* [20], indicating conserved compositional patterns despite the influence of environmental conditions [21]. However, the absolute dominance of *Ascomycota* within the fungal communities across different *P. helenae* populations, coupled with significant compositional differences among geographic populations (e.g., the near absence of *Mucoromycota* in the SL population) in this study, highlights the critical influence of species specificity and habitat heterogeneity on microbial colonization in rhizosphere. Members of Actinobacteria phylum can degrade complex organic matter and synthesize antibiotics [22,23], while species of Proteobacteria phylum can significantly affect the nitrogen uptake by plants [24]. Members of *Acidobacteria* phylum can promote nutrient absorption by plants, enhance their resistance to stress like drought and salinity, and enable them to survive in acidified soils [25,26]. Fungi of *Ascomycota* phylum, known for their cellulose-degrading properties, are commonly found in semi-arid regions and forest soils [27,28]. These dominant microbial groups likely play critical roles in nutrient cycling in rhizosphere soil, enabling *P. helenae* to adapt to the barren karst soils.

### 4.2. Functional Analysis of Key Microbial Groups and Their Ecological Adaptability

The geographical variations in the rhizosphere microbial community of *P. helenae* may be related to its functional characteristics. The fungal community in the MQ population was significantly enriched with ecologically important genera, such as *Geminibasidium*, *Trichoderma*, and *Cladophialophora* sp. Studies have indicated that *Geminibasidium* sp. can transform soil nitrogen into plant-absorbable forms and enhance plant growth by improving the availability of nutrients in soil [29]. Given the habitat characteristics of MQ population (i.e., high pH and low nitrogen and phosphorus content), the enrichment of such fungi may alleviate nutrient stress in karst drought areas through higher organic matter degradation and nutrient activation in soil. *Trichoderma* can improve the physicochemical properties of soil, promote the establishment and maintenance of beneficial microbial communities, and enhance crop resistance [30]. *Cladophialophora* sp. can also stimulate plant growth and control the incidence of certain plant diseases [31]. The MQ population demonstrated a significant enrichment of specific bacterial taxa, including Firmicutes and the unclassified family norank_f_*Xanthobacteraceae*. Members of the phylum Firmicutes are known to enhance plant tolerance to abiotic stresses, such as drought, through mechanisms including osmotic regulation, ion homeostasis maintenance, and antioxidant production under extreme conditions [32,33]. Certain Firmicutes species also contribute to plant health by suppressing pathogenic growth or inducing systemic resistance [34]. Meanwhile, norank_f_*Xanthobacteraceae*, an unclassified lineage within the *Xanthobacteraceae* family, encompasses bacteria capable of nitrogen fixation and denitrification, processes vital for soil fertility and plant nutrition [35]. Therefore, the markedly enriched microbial taxa in the MQ population may function synergistically: *Geminibasidium* and unclassified *Xanthobacteraceae* likely improve nitrogen availability, while *Trichoderma*, *Cladophialophora*, and Firmicutes contribute to microecological stability via pathogen antagonism and plant defense priming. This “functionally modular” community assembly pattern may be the key to the survival of *P. helenae* in low-nutrient habitats.

The LG population exhibited significant enrichment of *Patescibacteria* and *Gemmatimonadota* phyla. Both phyla consist of oligotrophic bacteria possessing highly efficient nutrient-scavenging capabilities at low nutrient concentrations [35,36]. Concurrently, the LG population showed significant enrichment of fungi, such as *Mortierella* and *Knufia* sp. *Mortierella* can promote plant growth and enhance salt tolerance [37], while *Knufia* sp. may facilitate the growth of desert plants in arid environments [38]. This functional profile aligns well with the karst-drought characteristics of the habitat of LG population in this study.

The rhizosphere fungal community of SL population exhibited a highly simplified structure, with overwhelming dominance of *Ascomycota* phylum. The significantly enriched genus Nigrospora includes several plant pathogens. For instance, *N. sphaerica* can induce leaf spot disease. This imbalanced community structure may be linked to the synergistic effects of high SOM and AN concentrations, which promote excessive proliferation of *Ascomycota* and suppress *Basidiomycota* possessing symbiotic functions [39]. Furthermore, the absence of *Mucoromycota* phylum in the community weakened the mycorrhizal network, potentially diminishing the capacity of pathogen competition and exclusion [6]. However, the SL population showed enrichment of the genus *Penicillium*, known for its disease-suppressive functions, likely as a response to potential pathogenic threats [40]. Concurrently, the SL population also showed enrichment of the fungal genus *Sirastachys*. The fungal species of this genus can degrade plant residues, prevent pathogen proliferation, and provide nutrients for plant growth [41]. The SL population was significantly enriched in specific bacterial taxa, including *Chloroflexi*, Cyanobacteria, and an uncultured bacterium affiliated with the genus *Pseudonocardia*. Cyanobacteria can directly furnish a vital nitrogen source for *P. helenae* via biological nitrogen fixation. Concurrently, *Chloroflexi* and *Pseudonocardia* are known to facilitate the decomposition of complex organic matter, thereby enhancing the release of essential mineral elements such as phosphorus and potassium, likely through mechanisms like organic acid secretion. The synergistic activities of these microorganisms are posited to improve nutrient acquisition efficiency in *P. helenae*, a trait of particular importance for slow-growing orchids sensitive to nutrient availability [33]. The natural habitat of *P. helenae* typically has specific environmental challenges, including nutrient poverty, seasonal water fluctuations, and unique soil composition. The enriched *Chloroflexi* and Cyanobacteria, especially the extremophiles within these groups, may further enhance of plants to such abiotic stressors.

Comprehensive research suggests that *P. helenae* may adapt to environmental changes, regulate its own growth, and maintain the survival and persistence of its population by assembling core rhizosphere microbial communities with different functional combinations. This may suggest that one of the key strategies of *P. helenae* to respond and adapt to the extreme karst environment [42]. In the future research, we will focus on elucidating the functional role of the rhizosphere microbiome as a potential key factor in the survival strategy of *P. helenae* in highly specialized environments. The filtering effect exerted by different habitats on the rhizosphere soil microorganisms of *P. helenae* results in significant differences among the rhizosphere microbial communities of distinct populations. Compared to the bacterial communities, fungal communities showed higher degree of variations in β-diversity, indicating that fungal communities in rhizosphere soil were more sensitive to environmental changes.

### 4.3. Relationships Between Microbial Communities and Soil Factors

SOM serves as a vital source of carbon and energy for soil microorganisms, while AN is a key component of nitrogen sources [43]. Environmental factor association models (db-RDA, VPA) revealed that SOM and AN were the key drivers shaping the microbial communities in the rhizosphere soil of *P. helenae*. Together, they explained 49.87% and 16.39% of variations in the bacterial and fungal communities. SOM and AN caused significant increase in the abundance of taxa, such as *Ascomycota* and unclassified-k-Fungi. Unclassified-k-Fungi, representing understudied fungi, may include species that are uniquely adapted to specific combinations of SOM and AN. Consequently, their abundance increased significantly in the environment rich in SOM and AN. Ascomycetes are typically saprotrophs that are capable of decomposing complex organic matter. Thus, the carbon and energy provided by SOM likely favored this group [44]. Simultaneously, the presence of AN possibly enhanced the organic matter decomposition capacity of *Ascomycota* fungi, leading to its higher abundance in rhizosphere soil [45]. The synergistic effects of SOM and AN enhanced the abundance of *Chloroflexota*. This phylum contains phototrophic bacteria capable of photosynthetic carbon fixation under low-light conditions in karst fissures [46]. Additionally, species of this phylum can participate in the decomposition and transformation of organic matter, thereby significantly influencing the carbon and nutrient cycling in soil [47,48]. Therefore, increased SOM provided the members of *Chloroflexota* with abundant carbon sources, while AN fulfilled their nitrogen requirements. Thus, SOM and AN were positively associated with the growth of bacteria belonging to *Chloroflexota*. Conversely, SOM and AN showed a negative correlation with the growth of bacterial groups, such as *Myxococcota, Enterobacterota*, *Bacillota*, and *Methylomirabilota.* This may be attributed to the better adaptability of these groups to oligotrophic or specific nutrient conditions. For instance, *Methylomirabilota* exhibits a competitive advantage in environments with low concentrations of organic matter and nitrogen [49].

Soil pH is a key factor influencing the structure and function of microbial communities, and different microbial groups may exhibit distinct responses to pH [50]. The results of this study showed negative correlations of soil pH with most bacterial phyla. This observation suggests that most bacteria in the rhizosphere of *P. helenae* may show better adaptability to acidic environments. Notably, the correlations of pH with *Acidobacteriota* and *Gemmatimonadota* reached significant or highly significant levels. Research has found that certain microbes belonging to *Acidobacteriota* phylum can participate in organic acid metabolism, thereby promoting mineral weathering and the release of phosphorus and potassium [51,52]. In contrast, pH showed positive correlations with *Actinomycetota* and Cyanobacteria phyla, indicating that these groups were more adaptable to neutral or alkaline environments. Cyanobacteria phylum includes photoautotrophs that typically grow well under alkaline conditions [49]. Under higher pH conditions, members of *Actinomycetota* phylum are beneficial for decomposition of organic matter and promotion of nutrient cycling [53]. Most *Ascomycota* fungi prefer neutral or slightly acidic environments [50]. However, in this study, high pH promoted the growth of *Ascomycota*. This indicates that certain microbes within this phylum have developed adaptive mechanisms for high-pH environments, which allows them to occupy this ecological niche [54].

Nitrogen exerts a significant influence on the structure and diversity of soil microbial communities [55]. Elevated nitrogen levels can enhance plant photosynthesis. The resulting increase in litterfall and carbon input provides additional nutrient sources for microorganisms [56]. Fungi preferentially utilize carbon from litter, which consequently enhances fungal community diversity [57]. Studies have indicated that the relative abundance of *Ascomycota* typically rises under high-nitrogen conditions, as the fungi of this phylum may utilize excess nitrogen resources more efficiently for their growth and reproduction [58]. On the other hand, fungi of *Basidiomycota* phylum generally play crucial roles in decomposing complex organic matter like lignin, and high nitrogen levels may alter their metabolic pathways or reduce their competitiveness [59]. *Mucoromycota* is a group of common saprophytic fungi in soil. The high-nitrogen environments may inhibit the growth of these fungi due to associated changes in pH or other physicochemical properties of soil [60]. In this study, the nitrogen-rich environment was significantly positively correlated with the growth of *Ascomycota*, while significantly negatively correlated with the growth of *basidiomycota*, *Mucoromycota* and related groups. This correlation may have reduced the proportion of symbiotic mycorrhizae in the rhizosphere soil community, which explains the enrichment of pathogens in the SL population. *Bacteroidota* and *Pseudomonadota* are common soil bacterial groups involved in nitrogen cycle processes like ammonification and denitrification. *Patescibacteria* characterized by smaller genomes, may have a competitive advantage in eutrophic environments [61]. Consequently, increased nitrogen levels in soil may elevate the abundance of these bacterial groups.

A significant positive correlation was observed between AP and AK. These factors collectively and significantly reduced the abundance of Actinobacteria, while increasing the abundance of *Patescibacteria* and *Nitrospirota* collectively and significantly or highly significantly. Combined with the relationships of bacterial α-diversity with *Actinomycetota*, *Patescibacteria*, and *Nitrospirota* phyla, this indicates that AP and AK were significantly positively correlated with the bacterial diversity in the rhizosphere soil of *P. helenae*. Similarly, AP and AK collectively and significantly or highly significantly increased the abundance of Chytridiomycota, indicating positive correlations of AP and AK with fungal diversity indices in the rhizosphere soil of *P. helenae*. Therefore, both AP and AK exerted a positive influence on the microbial α-diversity in rhizosphere of *P. helenae*.

The collective negative effects of AP and AK on *Actinomycetota* abundance may be attributed to the high levels of phosphorus and potassium, which can inhibit the growth of *Actinomycetota* or promote competition with the other microbial groups [62]. Conversely, the significant or highly significant increase in the abundance of *Patescibacteria*, *Nitrospirota*, and Chytridiomycota caused by AP and AK may indicate that these phyla are better adaptable to environments rich in phosphorus and potassium or can utilize specific resources under such conditions [63]. Collectively, these results suggest that soil factors are important drivers of microbial community differentiation. This observation is consistent with the patterns reported for other *Paphiopedilum* species [12]. However, the potential influence of other unmeasured environmental variables cannot be ruled out.

### 4.4. Implications of the Conservation Strategies for P. helenae

Geographic divergence in the rhizosphere microbiome of *P. helenae* and its nutrient dependency revealed novel endangerment mechanisms beyond established anthropogenic threats, over-collection, habitat degradation, and karst nutrient limitations [64,65]. The nutrient-deficient MQ population could survive by enriching stress-tolerant taxa (e.g., *Trichoderma*, *Didymella*), while the nutrient-rich SL population faces an elevated risk of extinction due to microbial dysbiosis (e.g., pathogen proliferation, symbiont suppression). These observations suggest that microbial dysfunction is a critical driving factor for endangerment of *P. helenae*. *P. helenae* adapts to heterogeneous environments by enriching functionally complementary microbial groups (e.g., drought-tolerant bacteria paired with plant-growth promoters, or pathogens counterbalanced by antagonistic microbes). This strategy likely results from the co-evolutionary adaptation of microbes. Tailored conservation strategies should be implemented based on population-specific microbial community structures: (1) The LG population, with high microbial diversity in N/P-rich rhizosphere soils, can be used as a resilient source population for restoration; (2) The MQ population requires vigilance against fungal diversity loss induced by habitat degradation, which can be mitigated by introducing saprophytic consortia or humus in the rhizosphere soil; (3) The SL population requires prioritized pathogen containment via dynamic monitoring and deployment of native biocontrol agents. Functional validation of keystone microbes (e.g., *Trichoderma*, *Knufia* sp.) and development of a core microbiome are essential to enhance stress resilience of plants and advance microbe-assisted conservation frameworks for karst-endangered flora.

### 4.5. Limitations and Future Research

This study provided crucial insights into the potential connections between rhizosphere microorganisms and survival of *P. helenae* population. Still, several limitations of this research need to be acknowledged. Firstly, the conclusions are primarily based on inferential methods and correlation analyses, which reveal associations but do not establish causality. Future studies should employ mechanistic experiments, such as isolation of key microbes, inoculation trials, and functional verification to confirm the interactions between rhizosphere microorganisms and *P. helenae* populations. Secondly, sampling was conducted at a single time point in this study. Microbial community composition and orchid physiological traits are known to exhibit seasonal dynamics, which could not be captured in this research. Multi-temporal sampling across different seasons would provide a more comprehensive understanding of these fluctuations. Thirdly, this study focused primarily on soil properties. Further research incorporating microclimate data (e.g., humidity, temperature, light exposure) and temporal dynamics would clarify the relative contributions of these factors to microbial community assembly in the rhizosphere soil. Lastly, due to the endangered status of *P. helenae* and restrictions on non-destructive sampling of this plant species, detailed morphological and physiological data (e.g., photosynthetic rates, leaf area, nutrient content) were not available. Although general habitat characteristics and population estimates were included in the research, the lack of plant-level trait data was a significant constraint.

A key consideration in interpreting the results of this study was the taxonomic resolution offered by amplicon sequencing. The analysis was conducted at the genus level, as species-level assignment can be uncertain for both prokaryotes and eukaryotes. For bacteria, this is due to the high sequence conservation of the 16S rRNA gene among the species within the same genus and current limitations in the reference databases. For fungi, the ITS region provides higher resolution, but the database limitations and the prevalence of uncharacterized environmental sequences often impede confident species-level identification. In studies of rhizosphere microbial diversity, genus-level profiles are generally sufficient to capture overall community structure, responses to environmental factors, and major ecological patterns. Many high-quality studies have also primarily reported genus-level microbial community structure [66,67,68]. To ensure consistency in taxonomic resolution between bacterial and fungal datasets and to avoid overinterpretation of potentially erroneous species-level assignments, genus was adopted as the standard level of analysis in this study. This approach maximizes data reliability and reproducibility and is aligned with best practices in the field. Future studies should employ shotgun metagenomics or strain-level culturing techniques to elucidate the functional roles and dynamics of specific species within the rhizosphere microbial genera identified here.

## 5. Conclusions

This study investigated the structure and diversity of rhizosphere microbial communities of the endangered orchid *Paphiopedilum helenae* in its natural habitats within the karst region of Guangxi, China, providing a reference for future development of functional soil microbial communities in karst areas. The results revealed significant geographical divergence in microbial community assembly, with fungal communities exhibiting greater sensitivity to environmental gradients than bacterial communities. Actinobacteria and Proteobacteria dominated the bacterial communities, while *Ascomycota* and *Basidiomycota* were predominant among fungi. SOM and AN were identified as key environmental factors associated with the structure of the rhizosphere microbial community of *P. helenae*. Their synergistic correlation was linked to increased abundance of phototrophic *Chloroflexota*, which may enhance carbon fixation. In addition, high nitrogen conditions were correlated with a reduction in symbiotic microorganisms and a potential increase in pathogenic taxa. Distinct functional microorganisms were observed across different populations, suggesting that *P. helenae* may adapt to habitat pressure by assembling a functionally complementary core microbiome. Furthermore, Microbial dysbiosis might represent as a novel endangerment mechanism that requires further validation. Future conservation efforts for *P. helenae* warrant population-tailored strategies, prioritizing exploration of core microbiomes and screening of functional microbial strains adaptable to karst rocky desertification.

## Figures and Tables

**Table 1:** Wild population information of *Paphiopedilum helenae* in Longzhou County, Guangxi.

Population Number	Collection Location	Position	Altitude (m)	Grade of Slope (°)	Aspect of Slope	Number of Plants	Growth Condition
MQ	Mingqiang village in Longzhou County	Rhizosphere	450	70–80	Southwest	50	The plants grew well, mainly in groups of more than 10 plants
LG	LongGang village in Longzhou County	Rhizosphere	430	70–80	Northwest	50	The plants grew well, mainly in groups of more than 10 plants
SL	Sanlian Village in Longzhou County	Rhizosphere	520	80–90	Northeast	40	The plants grew well, showing scattered small populations of 1–3 plants

**Table 2:** Physicochemical properties of rhizosphere soil across different *Paphiopedilum helenae* populations.

Number	Potential of Hydrogen (pH)	Nitrogen (N) (g·kg^−1^)	Phosohorus (P) (g·kg^−1^)	Potassium (K) (g·kg^−1^)	Soil Organic Matter (SOM) (g·kg^−1^)	Ammonium Nitrogen (AN) (mg·kg^−1^)	Available Phosphorus (AP) (mg·kg^−1^)	Available Potassium (AK) (mg·kg^−1^)
MQ	7.96 ± 0.04 b	6.60 ± 0.04 b	0.42 ± 0.02 b	4.80 ± 0.29 a	126.4 ± 0.56 c	374.58 ± 3.76 c	8.43 ± 0.35 c	93.59 ± 4.26 c
LG	5.97 ± 0.03 c	14.99 ± 0.15 a	0.58 ± 0.03 a	4.76 ± 0.26 a	305.34 ± 14.92 b	1002.68 ± 15.43 b	58.84 ± 1.18 a	220.76 ± 8.20 a
SL	8.05 ± 0.01 a	14.94 ± 0.11 a	0.43 ± 0.02 b	4.56 ± 0.32 a	398.88 ± 2.81 a	1104.50 ± 26.05 a	14.54 ± 0.27 b	204.15 ± 7.49 b

Notes: Data are presented as mean ± standard deviation, with the mean calculated from triplicate measurements. MQ represents Mingqiang populations. LG represents Longgang populations. and SL represents Sanlian populations. Different lowercase letters indicate significant differences in soil physicochemical properties among different groups (*p* < 0.05). N: nitrogen. P: phosohorus. K: potassium. SOM: soil organic matter. AN: ammonium nitrogen. AP: available phosphorus. AK: available potassium.

**Table 3:** The abundance and diversity index of soil microbial community.

Population Number	Bacterial				Fungi			
	Ace Index	Chao Index	Shannon Index	Simpson Index	Ace Index	Chao Index	Shannon Index	Simpson Index
MQ	2651.18 ± 89.64 b	2643.49 ± 79.72 b	5.89 ± 0.13 b	0.0084 ± 0.0016 a	712.69 ± 5101.89 b	696.44 ± 114.26 b	3.47 ± 0.24 b	0.0673 ± 0.0123 a
LG	2977.86 ± 137.52 a	2912.71 ± 131.73 a	6.40 ± 0.06 a	0.0047 ± 0.0005 b	1024.98 ± 104.24 a	1015.37 ± 102.26 a	4.67 ± 0.22 a	0.0263 ± 0.0053 b
SL	2511.45 ± 138.92 b	2515.80 ± 120.60 b	6.09 ± 0.14 b	0.0074 ± 0.0012 a	1014.26 ± 110.21 a	1013.32 ± 107.67 a	4.90 ± 0.04 a	0.0182 ± 0.0018 b

Note: Different small letters in the same column indicate significant differences (*p* < 0.05).

## Data Availability

The raw data supporting the conclusions of this article will be made available by the authors on request.

## References

[1] Averyanov L. (1996). *Paphiopedilum helenae* (Orchidaceae)-new slipper orchid from the North Vietnam. Bot. J..

[2] Huang Y., Xue Y. (2007). Conservation status of *Paphiopedilum helenae* Aver., a newly recorded orchid in China. Acta Phytotaxon. Sin..

[3] Zeng S., Chen Z., Wu K., Duan J. (2010). Study on introduction and cultivation of *Paphiopedilum* distributed in China. Chin. Wild Plant Resour..

[4] Yetgin A. (2023). The dynamic interplay of root exudates and rhizosphere microbiome. Soil Stud..

[5] Pérez-Jaramillo J., Mendes R., Raaijmakers J. (2015). Impact of plant domestication on rhizosphere microbiome assembly and functions. Plant Mol. Biol..

[6] Mendes R., Garbeva P., Raaijmakers J. (2013). The rhizosphere microbiome: Significance of plant beneficial, plant pathogenic, and human pathogenic microorganisms. FEMS Microbiol. Rev..

[7] Wang J., Bao J., Su J., Li X., Chen G., Ma X. (2015). Impact of inorganic nitrogen additions on microbes in biological soil crusts. Soil Biol. Biochem..

[8] Li H., Dong T., Wang M. (2016). Effects of biochar on microbial communities and metabolic activity in rhizospheric soil of banana seedlings. J. Microbiol..

[9] Quan Q., Yang Y., Liang J., Yang L., Wu C., Xue B. (2016). Soil Microflora change during integrated protection cultivation of wheat-maize rotation. Chin. Agric. Sci. Bull..

[10] Tian L., An M., Liu F., Zhang Y. (2024). Fungal community characteristics of the last remaining habitat of three *Paphiopedilum* species in China. Sci. Rep..

[11] Tian L., An M., Wu M., Liu F., Zhang Y. (2023). Habitat ecological characteristics and soil fungal community structure of *Paphiopedilum* subgenus *Brachypetalum* Hallier (Orchidaceae) plants in Southwest China. Plant Signal. Behav..

[12] Wu M. (2023). Rhizosphere Soil Microbial Diversity of *Paphiopedilum* Subgen. Brachypetalum Plants in China. Master’s Thesis.

[13] Zhang Z. (2023). Study on the Endangered Mechanism of Key Microorganisms in the Root-Endophytic and Rhizosphere of Four Extremely Small Populations of *Paphiopedilum* Plant Populations. Master’s Thesis.

[14] Zheng Z., Zhou Y., Huang W., Chen X., Xue X., Wu B., Peng J., Mo J., Zhang Q. (2024). Bacterial and fungal diversity in the old tea plant ecosystem of Camellia sinensis ‘Fujian Shuixian’ cultivated in Gujing. Acta Microbiol. Sin..

[15] Xu L., Zhang Y., Zhou M., Li J., Gao L. (2019). Diversity and ecological function of root-associated fungi in three *Cypripedium* species. Microbiol. China.

[16] Wen D., Yang N., Yang M. (2016). Effects of re-vegetation on soil microbial functional diversity in purple soils at different vegetation stages on sloping-land in Hengyang, Hunan Province, China. Chin. J. Appl. Ecol..

[17] Berendsen R.L., Pieterse C.M.J., Bakker P.A.H.M. (2012). The rhizosphere microbiome and plant health. Trends Plant Sci..

[18] Zhang L., Wang Q., Zhou Q., Yin Y., Qi J., Zhou Y. (2010). Endangered plant Diplandrorchis of the growth of soil microorganisms. North. Hortic..

[19] Zhou Q., Sun D., Li H., Yue J., Chen X., Qu B., Zhang L. (2020). Study on bacterial diversity in rhizosphere soil of rare and endangered species *Diplandrorchis sinica*. J. Shenyang Agric. Univ..

[20] Tan X., Yang X., Sun X., Zhou Y., Hu S., Yuan C., Shi Z. (2023). Analysis of fungal communities in roots and root-associated soil of *Nervilia fordii* from karst areas of Guangxi. Guihaia.

[21] Shen C., Xiong J., Zhang H., Feng Y., Lin X., Li X., Liang W., Chu H. (2013). Soil pH drives the spatial distribution of bacterial communities along elevation on Changbai Mountain. Soil Biol. Biochem..

[22] Teregulova G., Sineva O., Markelova N., Sadikova V., Uvarov G., Kovalenko M., Manucharova N. (2023). Evaluation of chitinolytic and antibiotic activity of *Streptomyces avidinii* Ina 01467 and *Micromonospora aurantiaca* INA 01468. Eurasian Soil Sci..

[23] Gupta A., Singh D., Singh S.K., Singh V.K., Singh A.V., Kumar A. (2019). Role of actinomycetes in bioactive and nanoparticle synthesis. Role of Plant Growth Promoting Microorganisms in Sustainable Agriculture and Nanotechnology.

[24] Johnston-Monje D., Lundberg D.S., Lazarovits G., Reis V.M., Raizada M.N. (2016). Bacterial populations in juvenile maize rhizospheres originate from both seed and soil. Plant Soil.

[25] Wang G., Wang L., Ma F. (2022). Effects of earthworms and arbuscular mycorrhizal fungi on improvement of fertility and microbial communities of soils heavily polluted by cadmium. Chemosphere.

[26] Kielak A.M., Cipriano M.A.P., Kuramae E.E. (2016). *Acidobacteria* strains from subdivision 1 act as plant growth-promoting bacteria. Arch. Microbiol..

[27] Bastida F., Hernández T., Albaladejo J., García C. (2013). Phylogenetic and functional changes in the microbial community of long-term restored soils under semiarid climate. Soil Biol. Biochem..

[28] Gil-Martínez M., López-García Á., Domínguez M.T., Kjøller R., Navarro-Fernández C.M., Rosendahl S., Marañón T. (2021). Soil fungal diversity and functionality are driven by plant species used in phytoremediation. Soil Biol. Biochem..

[29] Rei K., Hiroyuki N., Kazuhikosan N. (2021). Composition for Promoting Plant Growth and Application Thereof. CN Patent.

[30] Chen Y. (2019). Effects of Different Nitrogen Fertilizers Combined with *Trichoderma* on Growth and Nutrient Utilization of Cucumis Melo. Master’s Thesis.

[31] Qin L., Chen Y., Xie L., Zhang Y., Nong Q., Long Y., Zeng F. (2023). *Cladophialophora* sp. ms2 and Its Application. CN Patent.

[32] Gao L., Huang Y., Liu Y., Mohamed O.A.A., Fan X., Wang L., Li L., Ma J. (2022). Bacterial Community Structure and Potential Microbial Coexistence Mechanism Associated with Three Halophytes Adapting to the Extremely Hypersaline Environment. Microorganisms.

[33] Tiwari P., Bose S.K., Park K.-I., Dufossé L., Fouillaud M. (2024). Plant-Microbe Interactions under the Extreme Habitats and Their Potential Applications. Microorganisms.

[34] Vimal S.R., Singh J.S., Kumar A., Prasad S.M. (2024). The plant endomicrobiome: Structure and strategies to produce stress resilient future crop. Curr. Res. Microb. Sci..

[35] Lynch M.D.J., Neufeld J.D. (2015). Ecology and exploration of the rare biosphere. Nat. Rev. Microbiol..

[36] Herrmann M., Wegner C.-E., Taubert M., Geesink P., Lehmann K., Yan L., Lehmann R., Totsche K.U., Küsel K. (2019). Predominance of Cand. Patescibacteria in Groundwater Is Caused by Their Preferential Mobilization from Soils and Flourishing Under Oligotrophic Conditions. Front. Microbiol..

[37] Yuan Z., Sun H. (2015). *Montagnulaceae* Bacteria in Plants and Their Uses. CN Patent.

[38] Li X., He X., Hou L., Ren Y., Wang S., Su F. (2018). Dark septate endophytes isolated from a xerophyte plant promote the growth of *Ammopiptanthus mongolicus* under drought condition. Sci. Rep..

[39] Huang Q., Wang B., Shen J., Xu F., Li N., Jia P., Jia Y., An S., Amoah I.D., Huang Y. (2024). Shifts in C-degradation genes and microbial metabolic activity with vegetation types affected the surface soil organic carbon pool. Soil Biol. Biochem..

[40] Deng M. (2007). Screening for Antimicrobial Endophytes and Optimization of Its Ferment Factor. Master’s Thesis.

[41] Ai Y., Xie T., Liu J., Lan S., Peng D., Zhang Q. (2019). Community structure and biological function of the root symbiotic fungi of *Arundina graminifolia*. Mycosystema.

[42] Jiang C., Liu Y., Li H., Zhu S., Sun X., Wu K., Shui W. (2022). The characterization of microbial communities and associations in karst tiankeng. Front. Microbiol..

[43] Yanardağ I.H., Zornoza R., Bastida F., Büyükkiliç-Yanardağ A., García C., Faz A., Mermut A.R. (2017). Native soil organic matter conditions the response of microbial communities to organic inputs with different stability. Geoderma.

[44] Manici L.M., Caputo F., Fornasier F., Paletto A., Ceotto E., De Meo I. (2024). *Ascomycota* and *Basidiomycota* fungal phyla as indicators of land use efficiency for soil organic carbon accrual with woody plantations. Ecol. Indic..

[45] Gu Y., Chen X., Shen Y., Chen X., He G., He X., Wang G., He H., Lv Z. (2023). The response of nutrient cycle, microbial community abundance and metabolic function to nitrogen fertilizer in rhizosphere soil of Phellodendron chinense Schneid seedlings. Front. Microbiol..

[46] Thiel V., Fukushima S.-I., Kanno N., Hanada S. (2019). Chloroflexi. Reference Module in Life Sciences.

[47] Lyu H., Sawada K., Zhong R., Kilasara M., Hartono A., Dahlgren R.A., Funakawa S., Watanabe T. (2024). Disentangling divergent factors controlling bacterial and fungal communities in topsoil and subsoil horizons across environmental gradients of tropical volcanic regions. CATENA.

[48] Chen J., Li F.-C., Jia B., Gang S., Li Y., Mou X.M., Kuzyakov Y., Li X.G. (2024). Regulation of soil nitrogen cycling by shrubs in grasslands. Soil Biol. Biochem..

[49] Wu J., Qi L., Huang T., Wang J., Sun Q. (2023). A short period of revegetation and fertilization increased the nutrients, enzyme activities, and bacterial community diversity in backfill soils. Appl. Soil Ecol..

[50] Rousk J., Brookes P.C., Bååth E. (2010). Investigating the mechanisms for the opposing pH relationships of fungal and bacterial growth in soil. Soil Biol. Biochem..

[51] Sarikhani M.R., Khoshru B., Oustan S. (2016). Efficiency of Some Bacterial Strains in Potassium Release from Mica and Phosphate Solubilization under In Vitro Conditions. Geomicrobiol. J..

[52] Serna Posso E.J., Sánchez de Prager M., Cisneros Rojas C.A. (2017). Organic acids production by rhizosphere microorganisms isolated from a Typic Melanudands and its effects on the inorganic phosphates solubilization. Acta Agron..

[53] Xu X., Wang J., Niu Y., Jiang W., Wang Y., Liu S., Wei W. (2024). 44-Years of Fertilization Altered Soil Microbial Community Structure by Changing Soil Physical, Chemical Properties and Enzyme Activity. J. Soil Sci. Plant Nutr..

[54] Shi Y., Li Y., Yang T., Chu H. (2021). Threshold effects of soil pH on microbial co-occurrence structure in acidic and alkaline arable lands. Sci. Total Environ..

[55] Liao L., Wang X., Wang J., Liu G., Zhang C. (2021). Nitrogen fertilization increases fungal diversity and abundance of saprotrophs while reducing nitrogen fixation potential in a semiarid grassland. Plant Soil.

[56] Zhang J., Li T., Jia J., Zhang J., Zhang F. (2021). Bacterial taxa and fungal diversity are the key factors determining soil multifunctionality in different cropping systems. Land Degrad. Dev..

[57] Qin Y., Pan X., Kubicek C., Druzhinina I., Chenthamara K., Labbé J., Yuan Z. (2017). Diverse Plant-Associated Pleosporalean Fungi from Saline Areas: Ecological Tolerance and Nitrogen-Status Dependent Effects on Plant Growth. Front. Microbiol..

[58] Yu W., Hall S.J., Hu H., Dutta S., Miao Q., Wang J., Kang H. (2022). Chronic nitrogen deposition drives microbial community change and disrupts bacterial-fungal interactions along a subtropical urbanization gradient. Soil Biol. Biochem..

[59] Jiang S., Liu Y., Luo J., Qin M., Johnson N.C., Öpik M., Vasar M., Chai Y., Zhou X., Mao L. (2018). Dynamics of arbuscular mycorrhizal fungal community structure and functioning along a nitrogen enrichment gradient in an alpine meadow ecosystem. New Phytol..

[60] Tomazelli D., Klauberg-Filho O., Mendes S.D.C., Baldissera T.C., Garagorry F.C., Tsai S.M., Pinto C.E., Mendes L.W., Goss-Souza D. (2023). Pasture management intensification shifts the soil microbiome composition and ecosystem functions. Agric. Ecosyst. Environ..

[61] Zhang H., Degré A., De Clerck C., Li S., Lian J., Peng Y., Sun T., Luo L., Yue Y., Li G. (2024). Changes in bacterial community structure and carbon metabolism in sandy soil under the long-term application of chitin-rich organic material and attapulgite. Appl. Soil Ecol..

[62] Chen Q., Cao J., Zhang M., Guo L., Omidvar N., Xu Z., Hui C., Liu W. (2025). The role of soil chemical properties and microbial communities on Dendrocalamus brandisii bamboo shoot quality, Yunnan Province, China. Front. Microbiol..

[63] Deng S., Wu Y., Wu K., Fang L., Li L., Zeng S. (2020). Breeding characteristics and artificial propagation of 14 species of Wild Plant with Extremely Small Populations (WPESP) in China. Biodivers. Sci..

[64] Shi J., Chen H., An M., Zhang Y., Ye C., Wu J. (2022). Analyses on distribution characteristics and protection effect of wild *Paphiopedilum* in Guizhou Province. Guihaia.

[65] Wang J., Liu Y., Wang C., Zhang M., Wang X., Zhu L. (2015). Dynamic changes of rhizosphere soil microbial biomass and nutrition of different Cherry rootstocks. Acta Agric. Boreali-Occident. Sin..

[66] Akinola S., Ayangbenro A., Babalola O. (2021). Metagenomic Insight into the Community Structure of Maize-Rhizosphere Bacteria as Predicted by Different Environmental Factors and Their Functioning within Plant Proximity. Microorganisms.

[67] He X., Wang D., Jiang Y., Li M., Delgado-Baquerizo M., McLaughlin C., Marcon C., Guo L., Baer M., Moya Y.A.T. (2024). Heritable microbiome variation is correlated with source environment in locally adapted maize varieties. Nat. Plants.

[68] Thiergart T., Paloma D., Ellis T., Vannier N., Hacquard S. (2020). Root microbiota assembly and adaptive differentiation among European *Arabidopsis* populations. Nat. Ecol. Evol..

